# Mutation of Hashimoto’s Thyroiditis and Papillary Thyroid Carcinoma Related Genes and the Screening of Candidate Genes

**DOI:** 10.3389/fonc.2021.813802

**Published:** 2021-12-21

**Authors:** Lizhuo Zhang, Lingyan Zhou, Qingqing Feng, Qinglin Li, Minghua Ge

**Affiliations:** ^1^ Department of Head and Neck Surgery, Center of Otolaryngology-Head and Neck Surgery, Zhejiang Provincial People’s Hospital (People’s Hospital of Hangzhou Medical College), Key Laboratory of Endocrine Gland Diseases of Zhejiang Province, Hangzhou, China; ^2^ Second Clinical Medical College, Zhejiang Chinese Medical University, Hangzhou, China; ^3^ Department of Radiology (Ultrasound), Cancer Hospital of the University of Chinese Academy of Sciences (Zhejiang Cancer Hospital), Institute of Cancer and Basic Medicine (IBMC), Chinese Academy of Sciences, Hangzhou, China; ^4^ Chinese Academy of Sciences (CAS) Key Laboratory for Biomedical Effects of Nanomaterials and Nano Safety & Chinese Academy of Sciences (CAS) Center for Excellence in Nanoscience, National Center for Nanoscience and Technology of China, Beijing, China; ^5^ Scientific Research Department, Cancer Hospital of the University of Chinese Academy of Sciences (Zhejiang Cancer Hospital), Institute of Cancer and Basic Medicine (IBMC), Chinese Academy of Sciences, Hangzhou, China

**Keywords:** Hashimoto’s thyroiditis (HT), papillary thyroid carcinoma (PTC), genetic correlation, gene chip, somatic cell target region sequencing, Tag SNP

## Abstract

Clinical studies have shown similarities in the genetic background and biological functional characteristics between Hashimoto’s thyroiditis (HT) and papillary thyroid carcinoma (PTC), and that HT may increase risks of PTC. Here, we set to determine the gene expression specificity of HT and PTC by screening related genes or co-expressed genes and exploring their genetic correlation. Referencing the Oncomine database, HT-related genes were discovered to be expressed in many different types of thyroid cancer, such as TSHR that is highly expressed in thyroid cancer. An in-depth genetic analysis and verification of 35 cancer and paracancerous tissue pairs from patients with thyroid cancer, and 35 tissues and blood cells pairs from patients with Hashimoto’s thyroiditis was conducted. Gene chip technology research showed that TSHR, BACH2, FOXE1, RNASET2, CTLA4, PTPN22, IL2RA and other HT-related genes were all expressed in PTC, in which TSHR was significantly over-expressed in PTC patients sensitive to radioactive iodine therapy, while BACH2 was significantly under-expressed in these patients. The biologically significant candidate Tag SNP highlighted from HT-related genes was screened by the high-throughput detection method. Somatic mutations in patients with HT and PTC were detected by target region capture technique, and 75 mutations were found in patients with HT and PTC. The upstream regulatory factors of the different genes shared by HT and PTC were analyzed based on Ingenuity Pathway Analysis (IPA), and it was found that HIF-1α and PD-L1 could be used as important upstream regulatory signal molecules. These results provide a basis for screening key diagnostic genes of PTC by highlighting the relationship between some HT-related genes and their polymorphisms in the pathogenesis of PTC.

## Introduction

In recent years, a large amount of evidence has emerged suggesting that Hashimoto’s thyroiditis (HT) may increase the risk of papillary thyroid carcinoma (PTC) (1–3). Studies have found that the atypical hyperplasia of thyroid follicular epithelium in HT may be a precancerous lesion of PTC, given that it coincides with PTC’s expression profiles in terms of cytological and immunological markers (HBME-1, CK19, galectin-3, Cyclin-D1, TTF-1) (4, 5). This provides new evidence for the “Inflammation-mediated Carcinogenesis” of HT-PTC. Exploratory studies on the genetic or histological homogeneity of HT and PTC have found that the common BRAF-V600E mutation in PTC is related to HT (6) where the expression of BRAF protein in HT tissue is 2.2 times higher than that in normal thyroid tissue (7), in addition to being related to tumor size, extraglandular invasion, and pathological type. Such genetic and histological results of HT and PTC suggest that they are similar in some genetic backgrounds and biological functional characteristics, yielding potential discoveries in the occurrence and progression of thyroid cancer. This led us to investigate the specificity of gene expressions of HT and PTC by screening related genes or co-expression genes to explore their genetic correlation.

Our research group used bioinformatics to analyze the Oncomine database and found that HT-related genes (TSHR, BACH2, RNASET2, CTLA4, PTPN22, IL2RA) were expressed in different types of thyroid cancer. Among them, TSHR and RNASET2 were highly expressed in malignant tumors, especially TSHR in thyroid cancer, while BACH2, CTLA4, PTPN22, IL2RA, and other immune-related genes are expressed significantly lower in thyroid cancer. Focusing on these genes, we screened Tag SNP, detected and typed the patients’ Tag SNP using high-throughput detection to analyze their interrelationships, their polymorphisms, and the pathogenesis of PTC. Highlighting key pathogenic genes through this process will provide a molecular diagnostic basis for the screening of high-risk groups.

## Materials and Methods

### Human Tissues

In total, 35 pairs of thyroid cancer and paracancerous tissues, 35 pairs of tissues and blood cells of patients with Hashimoto’s thyroiditis provided by Zhejiang Cancer Hospital were analyzed. Written informed consent was obtained from all subjects, and this study was approved by the Ethics Committee of Affiliated Zhejiang Cancer Hospital, Hangzhou, China.

### Chemicals and Reagents

VariantBaits™ Target Enrichment Library Prep Kit (VBS95-1023-96D01; LC-Bio Technology, Co., Ltd. Hangzhou, China); VariantBaits™ Target Enrichment Purification Beads (NO. VBS95-0518-96D01; LC-Bio Technology, Co., Ltd. Hangzhou, China); VariantBaits™ Target Enrichment Probe (NO. VBS95-7336-96D01; LC-Bio Technology, Co., Ltd. Hangzhou, China); VariantBaits™ Target Enrichment Amplification Kit (NO. VBS95-1229-96D01; LC-Bio Technology, Co., Ltd. Hangzhou, China); VariantBaits™ Target Enrichment Wash Kit (NO. VBS95-0128C01-96D01; LC-Bio Technology, Co., Ltd. Hangzhou, China); VariantBaits™ Target Enrichment Hybridization Kit (NO. VBS95-0128C02-96D01; LC-Bio Technology, Co., Ltd. Hangzhou, China); VariantBaits™ Target Enrichment Adapter & Block Oligo A (NO. VBS95-0128C03A-96D01; LC-Bio Technology, Co., Ltd. Hangzhou, China); VariantBaits™ Target Enrichment Adapter & Block Oligo B (NO. VBS95-0128C03B-96D01; LC-Bio Technology, Co., Ltd. Hangzhou, China); DynabeadsMyOne Streptavidin T1 Magnetic Beads (NO. 65602; Invitrogen, US); High Sensitivity DNA Kit (NO. 5067-4626; Agilent, US); Qubit 1x dsDNA HS Assay Kit (NO. Q33230; Invitrogen, US); Platinum™ SYBR™ Green qPCR SuperMix-UDG (NO. 11733038; Invitrogen, US); Nuclease-Free Water (NO. AM9930; Ambion, US); Pipette head, 10 μL,200 μL,1000 μL, (Axygen,US); Eppendorf Tube,200 µL,600 µL,1.5 mL,2.0 mL (Axygen,US).

### Analysis of HT-Related Genes in Oncomine Database

Using international Oncomine data, it was confirmed that there were significant differences in HT-related genes among different differentiated thyroid cancer tissues.

### Gene Chip Verification

The expression of HT-related genes in PTC and its sensitivity to radioiodine therapy were studied using gene chip technology (Affymetrix HTA2.0) to verify the differential expression of the genes being focused on HT and PTC and to further analyze the correlation between HT-related genes and PTC.

### Analysis of Candidate Tag SNP

HaploView was used to select the candidate Tag SNP of HT-related gene (MAF > 0.5 position D > 0.8), and the bilateral 1000bp base of the gene was extended to screen for non-coding domain Tag SNP. Bioinformatics software (SNP Function Portal, SNP Hunter) was used to verify Tag SNP with biological functions such as transcriptional regulation, translation, and expression.

### Somatic Cell Target Region Sequencing

#### Analysis of Research Principle

This project uses the target region capture technique to analyze and count 35 pairs of thyroid cancer patients’ cancer and paracancerous tissues, as well as 35 pairs of Hashimoto’s thyroiditis patients’ tissues and blood cells to detect somatic mutations. It is fast with high accuracy and coverage, allowing it to become a commonly used technique in clinical and basic research that is suitable for the analysis of disease and cancer samples with large sample sizes, yielding higher accuracy in the detection of SNV, Indel, and other mutations (**Supplementary Figure S1**).

#### DNA Extraction and Quality Control

The concentration of DNA was determined by Qubit fluorescence quantitative instrument, the total amount of DNA was 200ng-1µg, the concentration was not lower than 10 ng/µL, OD260/280 ≥1.8, OD260/230 ≥1.5. The main band of electrophoresis was intact and there was no obvious degradation.

#### Construction of the Genomic Library

The genomic DNA was randomly segmented to an average length of 150-200bp, and the ends of the segmented DNA were flattened using terminal repair enzyme. Nucleobases of adenine (and others as required) were added to the 3’ ends of the flat terminal DNA, then the sequencing connector was connected to both ends of the DNA by ligation. The ligated product was amplified by PCR to obtain the genomic DNA library.

#### Hybridization and Capture

The constructed genomic library was hybridized with the target region-specific probe at 65°C for 24 hours. After the hybridization reaction was completed, the product was captured by streptavidin magnetic beads that was then amplified by PCR and purified by magnetic beads to obtain the final capture library.

(The process of building a VariantBaits™ Target Enrichment System database is shown in **Supplementary Figure S2**)

#### Sequence Analysis and Gene Screening

After the original sequencing sequence (Sequenced Reads) was obtained, the joint sequence, polyN, polyA, and other sequences were filtered, and the filtered valid reads sequencing data were passed through BWA (Burrows-Wheeler Aligner) (8). The results in BAM format were compared to the reference genome (9) before being put through SAMtools (10) to be put into order, and the repetitive sequences were marked with Picard. After labeling the repeat sequences, the CIGAR values provided by the BWA comparison results were re-aligned by INDEL, and the base quality values were corrected using Base Recalibration in the GATK software. The vep tool was used to annotate the structure of the mutation site and further sort out the candidate regions that caused the disease.

According to the position of single nucleotide polymorphism in the gene, it can be divided into gene non-coding region SNP, gene spacer region SNP (inter-gene region), and gene coding region SNP. Single nucleotide polymorphisms (SNP) that are not in the coding region of the gene may still affect gene splicing, transcriptional binding, messenger RNA degradation, or RNA sequences in the non-coding region. The location of the mutation has a great impact on subsequent functions.

Screened differential gene files were uploaded to the IPA (Ingenuity Pathway Analysis) platform for biological correlation analysis of genes: I The biological correlation network between genes was established based on the “Build-Path Explorer” module, and II was based on the “Core Analysis-Upstream Regulator” module. The upstream regulatory factors of genes were analyzed, and the regulatory mechanism network was established.

## Results

### Expression of HT-Related Genes in Thyroid Cancer

**Figure 1:**
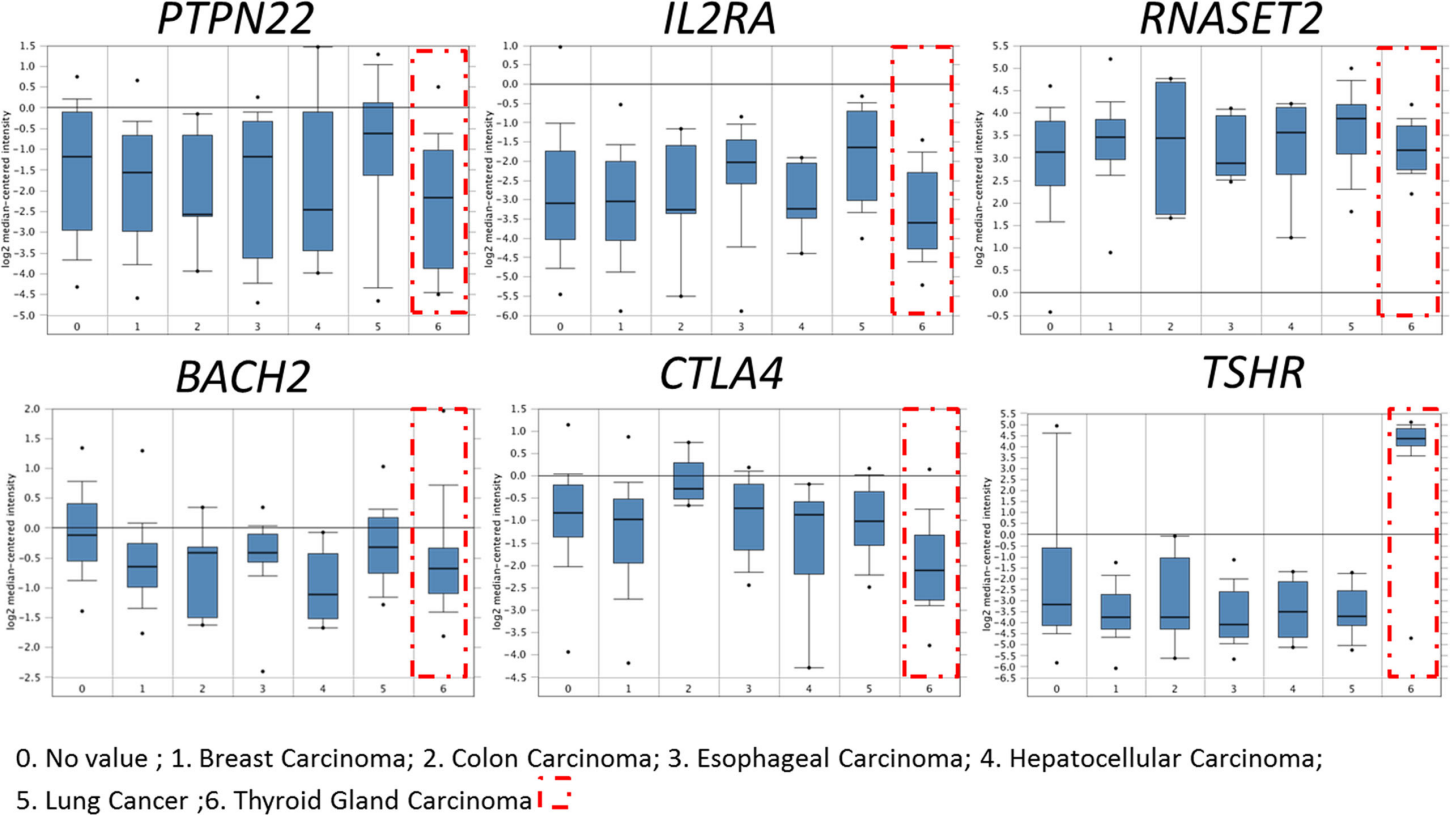
Expression of HT-related genes in papillary thyroid carcinoma. HT-related genes TSHR, BACH2, RNASET2, CTLA4, PTPN22 and IL2RA were expressed in thyroid cancer. Compared with normal tissues, immune-related genes BACH2, CTLA4, PTPN22, and IL2RA decreased significantly in thyroid cancer.

Using the bioinformatics method to analyze Oncomine database, it was found that HT-related genes (TSHR, BACH2, RNASET2, CTLA4, PTPN22, IL2RA) were also expressed in thyroid cancer, of which TSHR was especially highly expressed while immune-related genes such as BACH2, CTLA4, PTPN22, and IL2RA significantly decreased, as shown in **Figure 1**.

**Figure 2:**
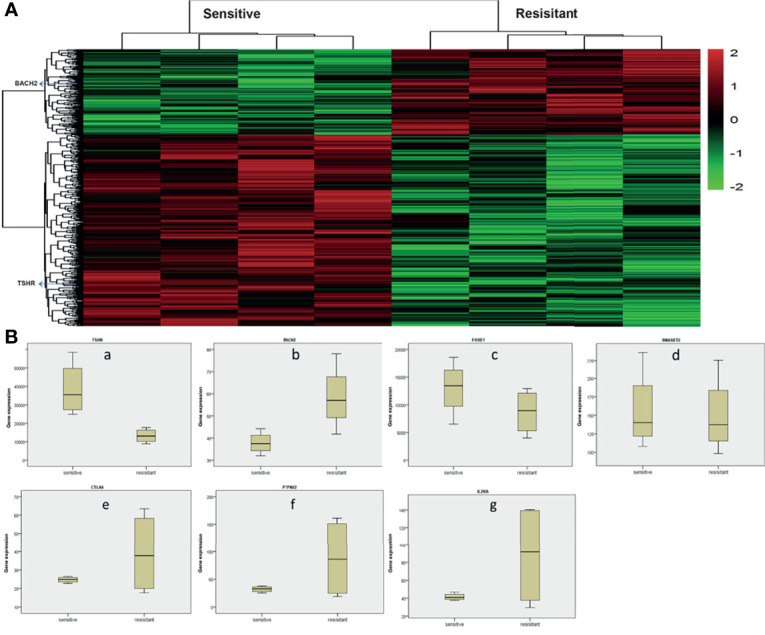
Analysis of whole gene expression profile in tumor tissues of radioiodine resistance and sensitive patients treated with radioactive iodine **(A)** Heat map of differential genes in tumor tissues of patients with resistance to radioactive iodine therapy and patients sensitive to radioactive iodine therapy. TSHR, BACH2, FOXE1, RNASET2, CTLA4, PTPN22, IL2RA, and other HT-related genes were all expressed in PTC. **(B)** Differential expression of HT-related genes in tumor tissues of radioiodine resistance and sensitive patients treated with radioactive iodine. Other than RNASET2, there were differences in the expression of other genes in tumor tissues of patients sensitive to radioiodine therapy and radioiodine resistance patients. Among them, the expression of TSHR was significantly higher in sensitive patients (p=0.005), and the expression of BACH2 was significantly lower in sensitive patients (p=0.037).

Gene chip technology (Affymetrix HTA2.0) was used to study the expression of HT-related genes in patients’ PTC tissues and their sensitivity to radioiodine therapy. The results showed that TSHR, BACH2, FOXE1, RNASET2, CTLA4, PTPN22, IL2RA, and other HT-related genes were all expressed in PTC. Other than RNASET2, there were differences in the expression of other genes in tumor tissues of patients sensitive to radioiodine therapy and radioiodine resistance patients. Among them, the expression of TSHR was significantly higher in sensitive patients (p=0.005), and the expression of BACH2 was significantly lower in sensitive patients (p=0.037), as shown in **Figure 2**. These results suggest that the above HT-related genes are expressed in PTC tissues and are of significance.

### Candidate Tag SNP of HT Related Genes

**Figure 3:**
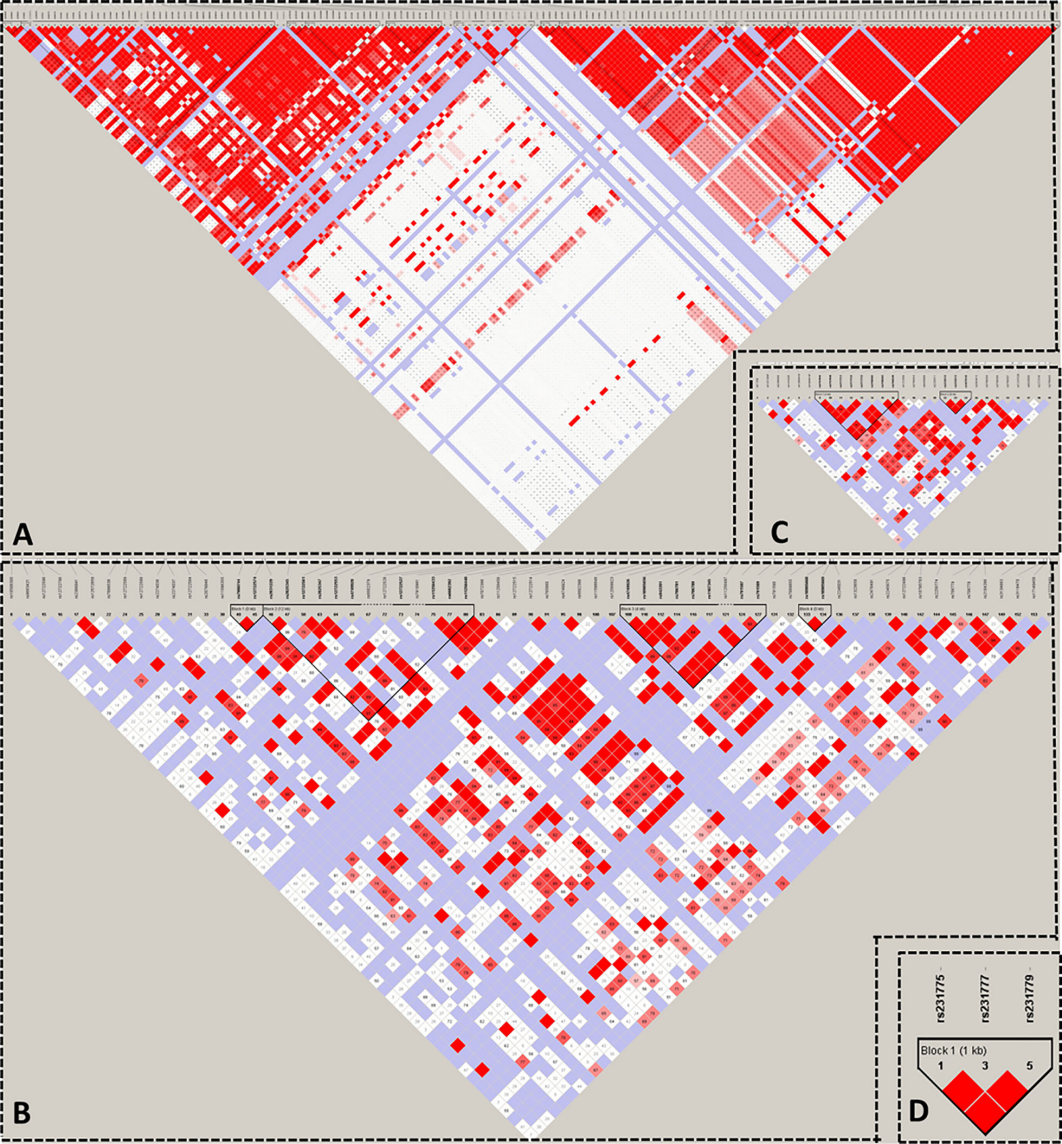
Linkage Disequilibrium Plot map of related gene Tag SNP. **(A)** TSHR; **(B)** IL2RA; **(C)** RNASET2; **(D)** CTLA4.

We used HaploView to select the candidate Tag SNP of HT-related gene (MAF>0.5; D>0.8) and extended the bilateral 1000bp bases of the gene as the screening of non-coding domain Tag SNP. A total of 2125 Tag SNP were screened. Bioinformatics software (SNP Function Portal, SNP Hunter) was used to verify Tag SNP with transcriptional regulation, translation and expression, and other biological functions. Biologically significant Tag SNP screening of TSHR, IL2RA, RNASET2, CTLA4, and PTPN22 genes have been completed totaling 16 candidate Tag SNPs. The Tag SNP of FOXE1 and BACH2 genes reached 952 and 1089 respectively, and the screening work is still in progress, as shown in **Figure 3** and **Table 1**.

**Table 1:** Candidate Tag SNP of related genes with biological significance.

Gene	Known number of Tag SNP	Candidate Tag SNP	
		SNP	Functional meaning
TSHR	27	rs12885526	Associated with the risk of thyroid Grave’s disease (11)
RNASET2	16	rs2236313	Associated with vitiligo susceptibility (12)
IL2RA	32	rs942201	Associated with childhood acute lymphoblastic leukemia (13)
		rs12722561	Associated with multiple liver cirrhosis (14)
		rs791587	Associated with childhood acute lymphoblastic leukemia and breast cancer (13, 15)
		rs2104286	Associated with the risk of multiple cirrhosis, type I diabetes, autoimmune Addison’s disease, thyroid exophthalmos and the progression of rheumatoid arthritis (16–21)
		rs2256774	Associated with multiple liver cirrhosis (21)
		rs706779	Associated with vitiligo susceptibility (22)
		rs706778	Associated with ulcerative colitis (23)
		rs3118470	Associated with hyperthyroidism (24)
		rs3134883	It is related to the susceptibility to vitiligo in the Han population (25)
CTLA4	2	rs231779	Associated with hyperthyroidism (26)
		rs231777	Associated with the risk of thyroid Grave’s disease (27)
PTPN22	7	rs1217395	Associated with the risk of type I diabetes (28)
		rs1217414	Associated with the risk of psoriasis (29)

### Specific Mutations in Patients With HT

This study included 35 pairs of inflammatory tissues from HT patients and their control samples, as well as 35 pairs of PTC patients and their paracancerous tissues. The aim was to identify disease-related sites. A total of 100 somatic mutations occurred in all HT and PTC patients, including a total of 1741 mutations in SNP and InDel. The main types of mutations were synonymous, nonsense, exon, and intron shear site, missense, frameshift, intron, 5 ‘regulatory region, and 3’ tail intergene region mutations.

The statistics of mutation sites in patients with papillary thyroid carcinoma and Hashimoto’s thyroiditis showed that there were 484 mutations in patients with Hashimoto’s thyroiditis and 92 corresponding genes in patients without mutations in PTC patients, which were arranged according to the location and impact of the mutation (**Table 2** and **Supplementary Figure S3**).

**Table 2:** Location of mutation in patients with Hashimoto’s thyroiditis.

Consequence	Proportion	Number of mutation sites
synonymous_variant	15.3%	74
3_prime_UTR_variant	29.1%	141
5_prime_UTR_variant	11.4%	55
intron_variant	5.6%	27
downstream_gene_variant	2.5%	12
missense_variant	24.2%	117
frameshift_variant	1.4%	7
inframe_deletion	0.2%	1
upstream_gene_variant	1.4%	7
splice_region_variant&synonymous_variant	0.2%	1
NA	7.6%	37
stop_gained	0.4%	2
splice_region_variant&5_prime_UTR_variant	0.2%	1
missense_variant&splice_region_variant	0.4%	2
Total	100%	484

NA, Not Available.

Re-screening of 150 mutation sites that may have a greater impact was conducted and covered 58 genes in total (downstream_gene_variant, missense_variant, frameshift_variant, inframe_deletion, upstream_gene_variant, splice_region_variant & synonymous_variant, stop_gained, splice_region_variant & 5_prime_UTR_varian™issense_variant&splice_region_variant). Among them, there were 4 mutation sites of importance for HT-related genes: TSHR (1 individual cell mutation) and BACH2 (3 individual cell mutation). These were sorted according to the number of mutation sites in HT and the influence of mutation. Preliminary screening showed that there were 22 mutation sites in at least two HT patients and 16 corresponding genes in patients with HT as follows: TPO, SLC4A3, TP63, CD74, VEGFA, IL17A, SLC26A4, NOS3, TG, RET, MKI67, VDR, PSMD9, MUC16, XRCC1, AR.

### Specific Mutations in Patients With PTC

For PTC-specific mutations, our strategy was to count a total of 564 somatic mutation sites, including 88 genes unique to PTC patients. Then based on the location of the mutations (CONSEQUENCE), the narrowing down of statistical distribution for mutation site was attempted (**Table 3** and **Supplementary Figure S4**).

**Table 3:** Location of mutation in patients with PTC.

Consequence	Proportion	Number of mutation sites
3_prime_UTR_variant	42.7%	241
5_prime_UTR_variant	9.9%	56
downstream_gene_variant	3.0%	17
frameshift_variant	1.8%	10
inframe_deletion	0.4%	2
intron_variant	3.4%	19
missense_variant	23.9%	135
missense_variant&splice_region_variant	1.1%	6
splice_region_variant&synonymous_variant	0.4%	2
synonymous_variant	12.4%	70
upstream_gene_variant	1.1%	6
Total	100.0%	564

According to the mutation locations that yielded greater impact, only 178 loci covering a total of 49 genes were screened (MUC16, HLA-DRB5, NDUFB2, MKI67, XRCC1, IL6R, SOAT1, FAM129A, SLC4A3, KDR, CD74, CDKN1A, ROS1, BRAF, TG, CD44, CCND1, TP53, BIRC5, PIAS3, TPO, LOC105373805, CTLA4, MMRN1, TERT, EDN1, TNF, VEGFA, BACH2, RNASET2, SLC26A4, NOS3, RET, CD4, LRRK2, FLT1, LGALS3, IGHE, PIAS1, CIITA, CDH1, SLC4A1, ICAM1, TGFB1, CEACAM5, BAX, E2F1, CSF2RB, AR).

The screening strategy was to retain the following locations or affected mutation sites: splice_region_variant & synonymous_variant, missense_variant, downstream_gene_variant, frameshift_variant, upstream_gene_variant, missense_variant & splice_region_variant, inframe_deletion. Among them, there were 7 mutation sites of importance for HT-related genes [CTLA4 (1 site), BACH2 (3 sites), and RNASET2 (3 sites)] where at least two patients with thyroid cancer experienced mutations. For thyroiditis patients with no mutation, the number of loci was 34 with 19 corresponding genes, and the genes were as follows: MUC16, HLA-DRB5, NDUFB2, MKI67, XRCC1, IL6R, SOAT1, FAM129A, SLC4A3, KDR, CD74, CDKN1A, ROS1, BRAF, TG, CD44, CCND1, TP53, BIRC5.

### Mutations in Patients With HT and PTC

Under effects of genetic and environmental factors, long-standing Hashimoto thyroiditis promotes thyroid malignant transformation, which eventually leads to papillary thyroid carcinoma. A total of 88 genes in 693 individual cell mutation sites of co-existing mutations were found in patients with HT and PTC (**Table 4** and **Supplementary Figure S5**).

**Table 4:** Location of mutations co-existing in HT and PTC.

Consequence	Proportion	Number of mutation sites
3_prime_UTR_variant	31.6%	219
5_prime_UTR_variant	8.9%	62
downstream_gene_variant	2.6%	18
frameshift_variant	2.5%	17
frameshift_variant&splice_region_variant	0.1%	1
inframe_deletion	1.4%	10
intron_variant	6.3%	44
missense_variant	25.5%	177
missense_variant&splice_region_variant	0.3%	2
splice_region_variant&synonymous_variant	0.3%	2
start_lost	0.1%	1
stop_gained,synonymous_variant	0.1%	1
synonymous_variant	18.8%	130
upstream_gene_variant	1.3%	9
Total	100.0%	693

Subsequently, 282 individual cell mutation sites of 56 candidate genes were screened according to the location of the mutation, and the genes were as follows: STAT1, NDUFB2, MUC16, CD44, CSF2RB, AR, HLA-DRB5, SLC26A4, VEGFA, FAM129A, KRT7, TP63, MMRN1, IL1B, FASLG, FLT1, TERT, CCND1, VDR, TPO, NOS3, IGF1, PIAS1, NGF, SLC4A3, BACH2, TSHR, CDH1, TGFB1, BAX, FOXE1, PTGS1, IFNG, BCL2, SLC5A5, PPARG, CD74, HLA-E, ROS1, MET, TAS2R38, TG, MKI67, CALCA, PGR, LRRK2, CIITA, MCL1, IL1R1, BRAF, RET, NOS2, BIRC5, XRCC1, FANCB, CD40LG. Notably, there were two mutation sites of importance on the HT-related gene BACH2, where the gene was also found on all PTC patients. Over 10 patients presented mutations on these two sites in addition to mutations on the following 7 genes: STAT1, NDUFB2, MUC16, CD44, CSF2RB, AR, HLA-DRB5.

### Analysis of Unique and Common Genes Between HT and PTC

**Figure 4:**
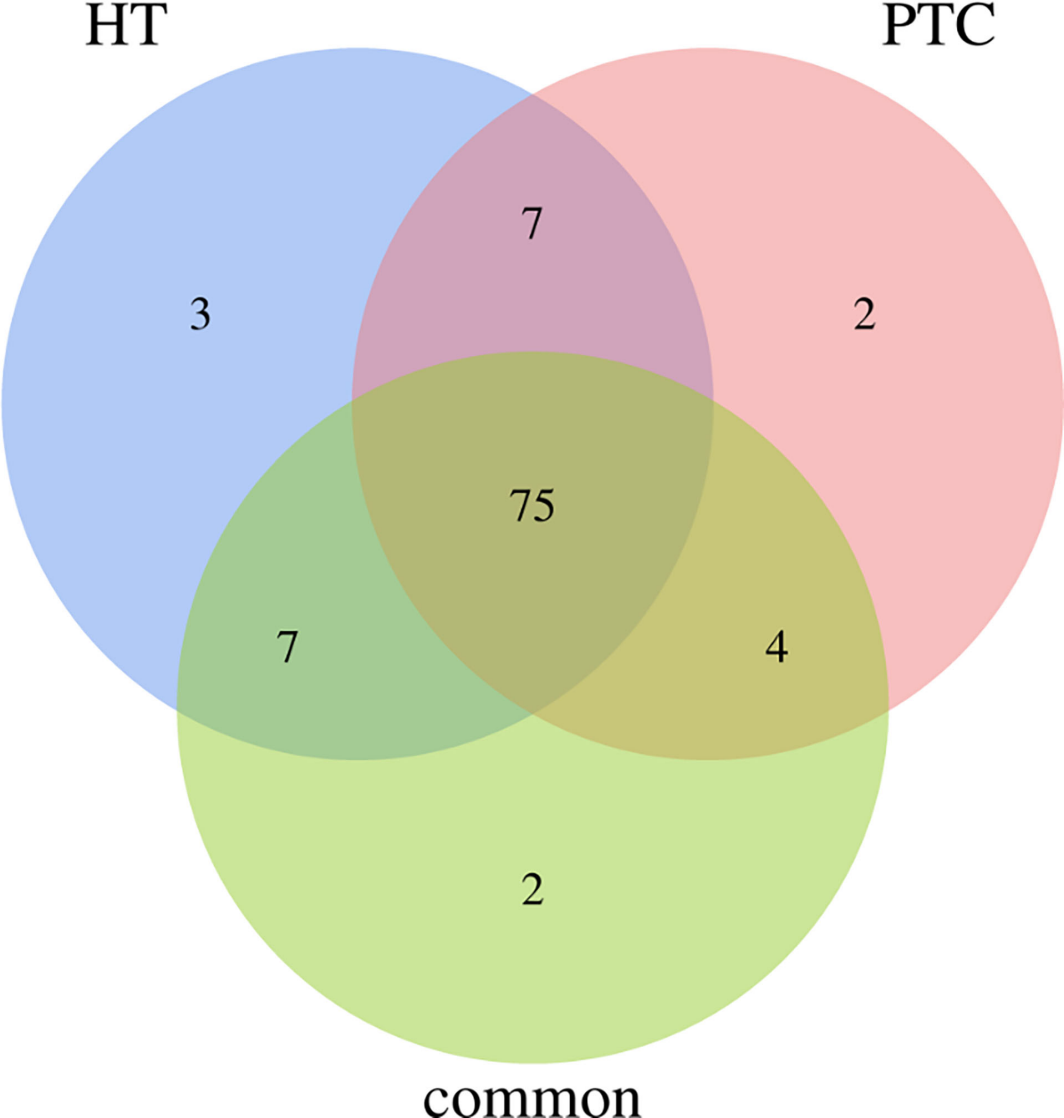
Venn diagram for genes that is common or specific to HT and PTC. 75% of the genes presented both cancer and inflammation.

Overall, 75% of all mutated genes identified were found to be present in all categories of cancer-specific, inflammation-specific, and when cancer and inflammation coexist, as shown in **Figure 4**. Genes from this 75% are as follows:

BAX, CTLA4, IGF1, SLC4A1, SOAT1, RET, BCL2, MUC16, SLC5A5, TGFB1, E2F1, MCL1, IL6R, PTGS2, TPO, STAT1, SLC4A3, TP63, NFKB1, CD74, HLA-DRB5, CDKN1A, VEGFA, IL17A, BACH2, SLC26A4, NOS3, TG, PTEN, MKI67, CCND1, PGR, CDKN1B, LRRK2, VDR, PSMD9, FLT1, TSHR, IGHE, CIITA, XRCC1, NELFCD, AR, CD40LG, TNFRSF25, NGF, FAM129A, CD8A, IL1R1, PPARG, KDR, MMRN1, TERT, F2R, EDN1, HLA-E, ROS1, RNASET2, MET, NDUFB2, BRAF, FOXE1, PTGS1, IL2RA, CD44, CD4, KRT7, PIAS1, DH1, STAT3, BIRC5, POLR2E, ICAM1, CSF2RB, FANCB.

**Figure 5:**
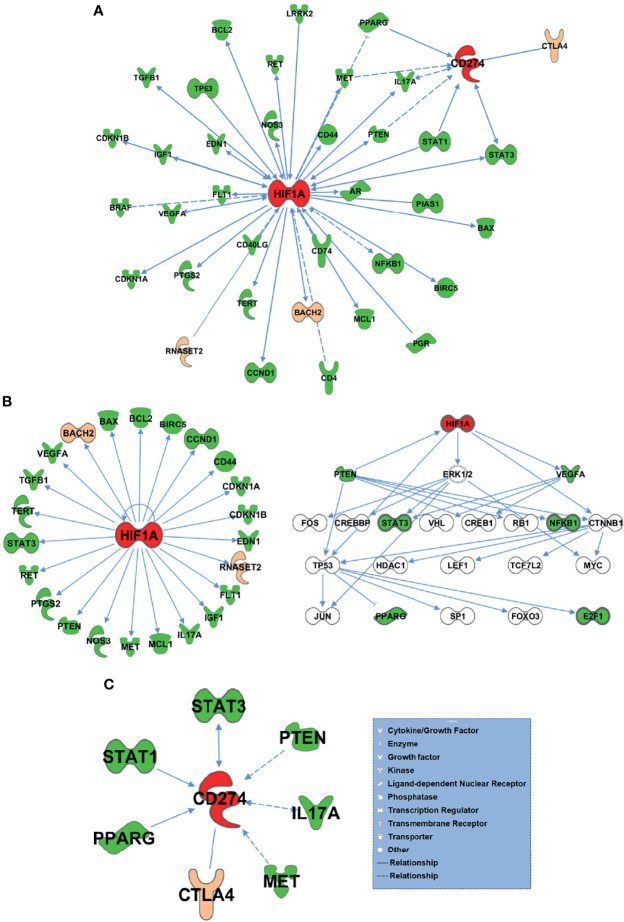
75 HT and PTC common differential genes and HIF-1α and PD-L1 bio related networks. **(A)** 75 common differential genes were biologically related to HIF-1α and PD-L1 genes. **(B)** The network of HIF-1α -related differential genes and their regulatory mechanisms. **(C)** PD-L1-related differential genes.

The 75 differential genes shared by HT and PTC were first uploaded to IPA (Ingenuity Pathway Analysis) software and set as subset A, while HIF-1α and PD-L1 (CD274) genes were set as subset B. Based on the database of the IPA system and the “Build-Path Explorer” module of the software, the biological correlation between subset A and B was analyzed. 75 common differential genes were biologically related to HIF-1α and PD-L1 genes, as shown in **Figure 5A**. Through the “Core Analysis-Upstream Regulator” module, the upstream regulatory factors of 75 common differential genes were analyzed, and it was found that HIF-1α and PD-L1 could be used as important upstream regulatory mechanisms. The network of HIF-1α -related differential genes and their regulatory mechanisms are shown in **Figure 5B**. PD-L1-related differential genes are shown in **Figure 5C**. Among the 75 differential genes, we selected BACH2 and RNASET2 related to HIF-1α, and CTLA4 related to PD-L1 as potential key molecules for further study.

On the GEO data Gene Expression Omnibus https://www.ncbi.nlm.nih.gov/geo/, the original file “GSE29315_RAW.tar” for human tissue gene expression microarray GSE29315 of HT and PTC could be downloaded. After decompressing, the original gene expression profile files of HT and PTC samples were selected (**Supplementary Table S1**).

Under R3.5.1, the original file of gene expression profile was read by affy package and corrected by the RMA method. The quantile of the corrected expression profile matrix was standardized by preprocessCore package. Expression profile matrix were annotated by ALL package and hgu95av2.db package to generate gene expression profile matrix files of HT and PTC samples (**Supplementary Figures S6**, **S7**).

## Discussion

Recent studies have found that the atypical hyperplasia of thyroid follicular epithelium in HT may be a precancerous lesion of PTC, given that it coincides with PTC’s expression profiles in terms of cytological and immunological markers (4, 5). This provides new evidence for the transformation of HT-PTC.

A few exploratory studies on the genetic or histological homogeneity of HT and PTC have suggested that BRAF-V600E mutation, which is very common in PTC, is not only related to tumor size, extraglandular invasion, and pathological type, but also HT (6), where the expression of BRAF protein was 2.2 times higher than that in normal thyroid tissue (7). Larson found that PI3K pathway - the key pathway of tumor progression especially in phosphorylated Akt, Akt1 and Akt2, was expressed in thyroid cancer and Hashimoto’s thyroiditis, but not in normal thyroid tissues (30). RET/PTC gene rearrangement in the ret tyrosine kinase domain can activate the activity of proto-oncogene ret, while existing family studies have found two specific ret rearrangements (RET/PTC1 and RET/PTC3 rearrangements) shared by HT and PTC tissues (31). In addition, the specific correlation of other tumor-related key molecules in HT and PTC has also been reported: compared with normal thyroid tissues or other thyroiditis tissues, in HT and PTC tissues, CD98 (involved in the transport of amino acids on the cell surface) was significantly lower expressed (32), p63 specifically expressed (33) and hOGG1 gene specifically deleted. All of these may be involved in the carcinogenic process of thyroid cancer (34). These genetic and histological specific correlation between HT and PTC suggest that HT and PTC are similar in some genetic background and biological functional characteristics and may be a key factor in the occurrence and progression of thyroid cancer, which opens a new perspective for the study of the relationship between HT and PTC. At present, genome-wide association studies (GWAS) and other studies have preliminarily identified genes associated with Hashimoto’s thyroiditis (35–37), including genes that encode for thyrotropin receptor (TSHR), BACH2 (BACH2), FOXE1 (FOXE1), HLA class I molecules (HLA class I region), ribonuclease 2A (RNASET2), cytotoxic T lymphocyte-associated antigen 4 (CTLA4), protein tyrosine phosphatase non-receptor type 22 (PTPN22), and interleukin-2 receptor α chain (IL2RA).

The above genes play a significant role in thyroid function, immune regulation, and tumor progression. 1) Thyroid specific gene: TSHR is a thyroid hormone receptor, which is highly related to autoimmune thyroid diseases; FOXE1 participates in thyroid morphogenesis, binds to the promoter region of thyroglobulin and thyroid peroxidase, and participates in the expression of thyroglobulin and thyroid peroxidase. 2) B cell regulatory gene: BACH2, which is expressed only during B cell maturation and helps control B cell development and antibody production (B cell functional changes may play an important role in AITD). 3) T cell regulatory genes: PTPN22 may be involved in T cell signal transduction and T cell receptor signal pathway; IL2RA in CD25^+^ regulatory T cells may have a down-regulating effect on T cell activity. HLA class I molecules play a key role in the formation of endogenous antigens so that they can be recognized by CD8^+^ T cells for immune response. 4) Other genes: ribonuclease 2A, the only member of the ribonuclease 2A protein family, may be involved in inhibiting carcinogenic processes.

Based on Somatic Cell Target Area Capture and IPA analysis, 75 genes were common in both cancer and inflammation. Among the 75 differential genes, we selected potential key molecules for further study including BACH2 and RNASET2 related to HIF-1α, along with CTLA4 that is related to PD-L1. And it was found that HIF-1α and PD-L1 can be used as important upstream regulatory mechanisms. HIF-1α, induced by hypoxia, plays important roles in the development and metastasis of many tumors, including PTCs, and is also associated with tumorigenesis or radiosensitivity (38, 39). Liu et al. demonstrated that HIF-1α was significantly higher in PTC than normal thyroid tissues (40), and the latest research showed that HIF-1α expression was not only upregulated in PTCs but was associated with high tumor-node-metastasis stage. HIF-1α can also bind to the promoter of Telomerase Reverse Transcriptase (TERT) and act as a potent transactivator, inducing autophagy thereby promoting PTC (41). Herein, we discovered that differential genes between HT and PTC were biologically related to HIF-1α and PD-1. Our results suggest HIF-1α is a signal that warns of high risks in HT evolving into PTC, and that HIF-1α, BACH2 and RNASET2 may serve as potential biomarkers of PTC.

The VariantBaits™ target region sequencing (Target Enrichment System) technology used in this paper can enrich the DNA of the protein-coding region of interest or specific sequences on the genome and carry out high-throughput sequencing on the Illumina second-generation sequencing platform. Compared with whole-genome sequencing, target region sequencing has many advantages, such as low cost, fast data analysis, high coverage depth, and high detection accuracy (42). However, systematic deviations could still be introduced by the sequencing instrument itself, thus impacting analysis downstream. For example, before correcting the reads of base quality value, we needed to retain bases with a mass value above Q25. Yet, the error rate of these bases with a mass value of Q25 is 1%, thus yielding a quality value of only Q20 and lowering the credibility of subsequent mutation detection. In the process of edge synthesis and sequencing, the error rate of the base at the end of reads is often higher than that at the beginning, and the quality value of AC is often lower than that of TG. Therefore, we used Base Recalibration in the GATK software to correct the base quality value, in hopes that the corrected sequence will be more uniform and reliable.

Molecular testing of mutation hotspots, rearrangements and gene expression using fine-needle aspiration cytology has been successfully used in the diagnosis and treatment of thyroid cancer (43, 44). As a MAPK-driven cancer, PTC showed two mutually exclusive actors with distinct signaling consequences: BRAFV600E and mutated RAS (45). Previous studies directly showed a high prevalence of BRAF mutations RAS mutations (46, 47). Consistent with previous studies, BRAF mutations were detected in PTC in this paper. In addition, we discovered new mutations in PTC along with 7 mutation sites of importance for HT-related genes CTLA4 (1 site), BACH2 (3 sites), and RNASET2 (3 sites). Compared with HT, PTC-specific mutant genes MUC16, HLA-DRB5, NDUFB2, MKI67, XRCC1, IL6R, SOAT1, FAM129A, SLC4A3, KDR, CD74, CDKN1A, ROS1, BRAF, TG, CD44, CCND1, TP53 and BIRC5 were detected. These genes have the potential to become markers for PTC diagnosis and even further enhance the care of these patients.

## Conclusion

In summary, we found 75 mutations in patients with HT and PTC and analyzed the upstream regulatory factors of the different genes shared by HT and PTC based on IPA. It was found that HIF-1α and PD-L1 could be used as important upstream regulatory mechanisms. These results provide a basis for screening key diagnostic genes of PTC by highlighting the relationship between some HT-related genes and their polymorphisms in the pathogenesis of PTC. HIF-1α and PD-L1 will thus be the main focus of our subsequent studies.

## Data Availability Statement

The datasets presented in this study can be found in Sequence Read Archive (SRA) https://www.ncbi.nlm.nih.gov/sra/?term=PRJNA786202.

## Ethics Statement

The studies involving human participants were reviewed and approved by Medical Ethics Committee of Zhejiang Cancer Hospital. The patients/participants provided their written informed consent to participate in this study.

## Author Contributions

MG and QL designed and supervised the study. LYZ collected clinical information of the patients. LZZ and LYZ prepared patient samples. LYZ, QF and LZZ analyzed the data. LZZ and QL wrote the manuscript. All the authors participated in the interpretation of the results and approved the final version of the manuscript.

## Funding

This study is supported by the National Natural Science Foundation of China (No.82173346), the Key Research and Development Program of Zhejiang Province (No.2021C03081), the Industry-University-Research Cooperative School-running Project of the Ministry of Education(No.202101160004), Science and Technology Program of traditional Chinese Medicine in Zhejiang Province(No. 2019ZZ004).

## Conflict of Interest

The authors declare that the research was conducted in the absence of any commercial or financial relationships that could be construed as a potential conflict of interest.

## Publisher’s Note

All claims expressed in this article are solely those of the authors and do not necessarily represent those of their affiliated organizations, or those of the publisher, the editors and the reviewers. Any product that may be evaluated in this article, or claim that may be made by its manufacturer, is not guaranteed or endorsed by the publisher.

## References

[1] Lun Y Wu X Xia Q Han Y Zhang X Liu Z. Hashimoto’s Thyroiditis as a Risk Factor of Papillary Thyroid Cancer May Improve Cancer Prognosis. Otolaryngol Head Neck Surg (2013) 148:396–402. doi: 10.1177/0194599812472426 23300224

[2] Kim KW Park YJ Kim EH Park SY Park DJ Ahn SH. Elevated Risk of Papillary Thyroid Cancer in Korean Patients With Hashimoto’s Thyroiditis. Head Neck (2011) 33:691–5. doi: 10.1002/hed.21518 21484918

[3] Dvorkin S Robenshtok E Hirsch D Strenov Y Shimon I Benbassat CA. Differentiated Thyroid Cancer Is Associated With Less Aggressive Disease and Better Outcome in Patients With Coexisting Hashimotos Thyroiditis. J Clin Endocrinol Metab (2013) 98:2409–14. doi: 10.1210/jc.2013-1309 23609834

[4] Ma H Yan J Zhang C Qin S Qin L Liu L. Expression of Papillary Thyroid Carcinoma-Associated Molecular Markers and Their Significance in Follicular Epithelial Dysplasia With Papillary Thyroid Carcinoma-Like Nuclear Alterations in Hashimoto’s Thyroiditis. Int J Clin Exp Pathol (2014) 7:7999–8007. 25550843 PMC4270599

[5] Chui MH Cassol CA Asa SL Mete O. Follicular Epithelial Dysplasia of the Thyroid: Morphological and Immunohistochemical Characterization of a Putative Preneoplastic Lesion to Papillary Thyroid Carcinoma in Chronic Lymphocytic Thyroiditis. Virchows Arch (2013) 462:557–63. doi: 10.1007/s00428-013-1397-1 23532502

[6] Kim YS Kim JS Bae JS Park WC. Clinical Implication of the BRAFV600E Mutation in Papillary Thyroid Carcinoma. World J Surg Oncol (2013) 11:99. doi: 10.1186/1477-7819-11-99 23687957 PMC3660263

[7] Derdas SP Soulitzis N Balis V Sakorafas GH Spandidos DA. Expression Analysis of B-Raf Oncogene in V600E-Negative Benign and Malignant Tumors of the Thyroid Gland: Correlation With Late Disease Onset. Med Oncol (2013) 30:336. doi: 10.1007/s12032-012-0336-3 23263826

[8] Li H Durbin R. Fast and Accurate Long-Read Alignment With Burrows-Wheeler Transform. Bioinformatics (2010) 26:589–95. doi: 10.1093/bioinformatics/btp698 PMC2828108 20080505

[9] Kent WJ Sugnet CW Furey TS Roskin KM Pringle TH Zahler AM. The Human Genome Browser at UCSC. Genome Res (2002) 12:996–1006. doi: 10.1101/gr.229102 12045153 PMC186604

[10] Li H Handsaker B Wysoker A Fennell T Ruan J Homer N. The Sequence Alignment/Map Format and SAMtools. Bioinformatics (2009) 25:2078–9. doi: 10.1093/bioinformatics/btp352 PMC2723002 19505943

[11] Bufalo NE Dos Santos RB Marcello MA Piai RP Secolin R Romaldini JH. TSHR Intronic Polymorphisms (rs179247 and rs12885526) and Their Role in the Susceptibility of the Brazilian Population to Graves’ Disease and Graves’ Ophthalmopathy. J Endocrinol Invest (2015) 38:555–61. doi: 10.1007/s40618-014-0228-9 25543543

[12] Quan C Ren Y Xiang L Sun L Xu A Gao X. Genome-Wide Association Study for Vitiligo Identifies Susceptibility Loci at 6q27 and the MHC. Nat Genet (2010) 42:614–8. doi: 10.1038/ng.603 20526339

[13] Chang JS Wiemels JL Chokkalingam AP Metayer C Barcellos LF Hansen HM. Genetic Polymorphisms in Adaptive Immunity Genes and Childhood Acute Lymphoblastic Leukemia. Cancer Epidem Biomar (2010) 19:2152–63. doi: 10.1158/1055-9965.EPI-10-0389 PMC3257312 20716621

[14] Maier LM Lowe CE Cooper J Downes K Anderson DE Severson C. IL2RA Genetic Heterogeneity in Multiple Sclerosis and Type 1 Diabetes Susceptibility and Soluble Interleukin-2 Receptor Production. PloS Genet (2009) 5:e1000322. doi: 10.1371/journal.pgen.1000322 19119414 PMC2602853

[15] Slattery ML Herrick JS Torres-Mejia G John EM Giuliano AR Hines LM. Genetic Variants in Interleukin Genes Are Associated With Breast Cancer Risk and Survival in a Genetically Admixed Population: The Breast Cancer Health Disparities Study. Carcinogenesis (New York) (2014) 35:1750–9. doi: 10.1093/carcin/bgu078 PMC4123645 24670917

[16] Wallace C Cutler AJ Pontikos N Pekalski ML Burren OS Cooper JD. Dissection of a Complex Disease Susceptibility Region Using a Bayesian Stochastic Search Approach to Fine Mapping. PloS Genet (2015) 11:e1005272. doi: 10.1371/journal.pgen.1005272 26106896 PMC4481316

[17] Heinonen MT Laine A Söderhäll C Gruzieva O Rautio S Melén E. GIMAP GTPase Family Genes: Potential Modifiers in Autoimmune Diabetes, Asthma, and Allergy. J Immunol (2015) 194:5885–94. doi: 10.4049/jimmunol.1500016 PMC4456634 25964488

[18] Fichna M Żurawek M Bratland E Husebye ES Kasperlik-Załuska A Czarnocka B. Interleukin-2 and Subunit Alpha of Its Soluble Receptor in Autoimmune Addison’s Disease – an Association Study and Expression Analysis. Autoimmunity (2014) 48:100–7. doi: 10.3109/08916934.2014.976628 25347332

[19] Knevel R de Rooy DPC Zhernakova A Gröndal G Krabben A Steinsson K. Association of Variants Inil2ra With Progression of Joint Destruction in Rheumatoid Arthritis. Arthritis Rheumatism (2013) 65:1684–93. doi: 10.1002/art.37938 23529819

[20] Chistiakov DA Chistiakova EI Voronova NV Turakulov RI Savost Anov KV. A Variant of the Il2ra / Cd25 Gene Predisposing to Graves’ Disease Is Associated With Increased Levels of Soluble Interleukin-2 Receptor. Scand J Immunol (2011) 74:496–501. doi: 10.1111/j.1365-3083.2011.02608.x 21815908

[21] Babron MC Perdry H Handel AE Ramagopalan SV Damotte V Fontaine B. Determination of the Real Effect of Genes Identified in GWAS: The Example of IL2RA in Multiple Sclerosis. Eur J Hum Genet (2012) 20:321–5. doi: 10.1038/ejhg.2011.197 PMC3283173 22085902

[22] Jin Y Birlea SA Fain PR Gowan K Riccardi SL Holland PJ. Variant of TYR and Autoimmunity Susceptibility Loci in Generalized Vitiligo. N Engl J Med (2010) 362:1686–97. doi: 10.1056/NEJMoa0908547 PMC2891985 20410501

[23] Bouzid D Amouri A Fourati H Marques I Abida O Tahri N. Polymorphisms in Theil2ra Andil2rb Genes in Inflammatory Bowel Disease Risk. Genet Test Mol Bioma (2013) 17:833–9. doi: 10.1089/gtmb.2013.0291 23972291

[24] Ban Y Tozaki T Taniyama M Nakano Y Ban Y Ban Y. Association of the Protein Tyrosine Phosphatase Nonreceptor 22 Haplotypes With Autoimmune Thyroid Disease in the Japanese Population. Thyroid (New York NY) (2010) 20:893–9. doi: 10.1089/thy.2010.0104 20615141

[25] Tang XF Zhang Z Hu DY Xu AE Zhou HS Sun LD. Association Analyses Identify Three Susceptibility Loci for Vitiligo in the Chinese Han Population. J Invest Dermatol (2013) 133:403–10. doi: 10.1038/jid.2012.320 22951725

[26] Zhao SX Pan CM Cao HM Han B Shi JY Liang J. Association of the CTLA4 Gene With Graves’ Disease in the Chinese Han Population. PloS One (2010) 5:e9821. doi: 10.1371/journal.pone.0009821 20352109 PMC2843719

[27] Li-qun G Wei Z Shuang-xia Z Lin Z Min-jia Z Bin C. Clinical Associations of the Genetic Variants of CTLA-4, Tg, TSHR, PTPN22, PTPN12 and FCRL3 in Patients With Graves’ Disease. Clin Endocrinol (2010) 72:248–55. doi: 10.1111/j.1365-2265.2009.03617.x 19438904

[28] Howson JMM Walker NM Smyth DJ Todd JA Type IDGC And TTID. Analysis of 19 Genes for Association With Type I Diabetes in the Type I Diabetes Genetics Consortium Families. Genes Immun (2009) 10:S74–84. doi: 10.1038/gene.2009.96 PMC2810493 19956106

[29] Smith RL Warren RB Eyre S Ke X Young HS Allen M. Polymorphisms in the PTPN22 Region Are Associated With Psoriasis of Early Onset. Brit J Dermatol (2008) 158:962–8. doi: 10.1111/j.1365-2133.2008.08482.x PMC2342636 18341666

[30] Larson SD Jackson LN Riall TS Uchida T Thomas RP Qiu S. Increased Incidence of Well-Differentiated Thyroid Cancer Associated With Hashimoto Thyroiditis and the Role of the PI3k/Akt Pathway. J Am Coll Surg (2007) 204:764–73, 773-5. doi: 10.1016/j.jamcollsurg.2006.12.037 17481480 PMC2430882

[31] Mechler C Bounacer A Suarez H Saint FM Magois C Aillet G. Papillary Thyroid Carcinoma: 6 Cases From 2 Families With Associated Lymphocytic Thyroiditis Harbouring RET/PTC Rearrangements. Br J Cancer (2001) 85:1831–7. doi: 10.1054/bjoc.2001.2187 PMC2364019 11747322

[32] Anderson CE Graham C Herriot MM Kamel HM Salter DM. CD98 Expression Is Decreased in Papillary Carcinoma of the Thyroid and Hashimoto’s Thyroiditis. Histopathology (2009) 55:683–6. doi: 10.1111/j.1365-2559.2009.03438.x 19922591

[33] Wirtschafter A Schmidt R Rosen D Kundu N Santoro M Fusco A. Expression of the RET/PTC Fusion Gene as a Marker for Papillary Carcinoma in Hashimoto’s Thyroiditis. Laryngoscope (1997) 107:95–100. doi: 10.1097/00005537-199701000-00019 9001272

[34] Royer MC Zhang H Fan CY Kokoska MS. Genetic Alterations in Papillary Thyroid Carcinoma and Hashimoto Thyroiditis: An Analysis of Hogg1 Loss of Heterozygosity. Arch Otolaryngol Head Neck Surg (2010) 136:240–2. doi: 10.1001/archoto.2010.20 20231640

[35] Simmonds MJ. GWAS in Autoimmune Thyroid Disease: Redefining Our Understanding of Pathogenesis. Nat Rev Endocrinol (2013) 9:277–87. doi: 10.1038/nrendo.2013.56 23529038

[36] Chu X Pan CM Zhao SX Liang J Gao GQ Zhang XM. A Genome-Wide Association Study Identifies Two New Risk Loci for Graves’ Disease. Nat Genet (2011) 43:897–901. doi: 10.1038/ng.898 21841780

[37] Cooper JD Simmonds MJ Walker NM Burren O Brand OJ Guo H. Seven Newly Identified Loci for Autoimmune Thyroid Disease. Hum Mol Genet (2012) 21:5202–8. doi: 10.1093/hmg/dds357 PMC3490518 22922229

[38] Covello KL Simon MC Keith B. Targeted Replacement of Hypoxia-Inducible Factor-1alpha by a Hypoxia-Inducible Factor-2alpha Knock-in Allele Promotes Tumor Growth. Cancer Res (2005) 65:2277–86. doi: 10.1158/0008-5472.CAN-04-3246 15781641

[39] Evens AM Schumacker PT Helenowski IB Singh AT Dokic D Keswani A. Hypoxia Inducible Factor-Alpha Activation in Lymphoma and Relationship to the Thioredoxin Family. Br J Haematol (2008) 141:676–80. doi: 10.1111/j.1365-2141.2008.07093.x PMC2894542 18422776

[40] Liu YM Ying SP Huang YR Pan Y Chen WJ Ni LQ. Expression of HIF-1alpha and HIF-2alpha Correlates to Biological and Clinical Significance in Papillary Thyroid Carcinoma. World J Surg Oncol (2016) 14:30. doi: 10.1186/s12957-016-0785-9 26846782 PMC4743326

[41] Song H Chen X Jiao Q Qiu Z Shen C Zhang G. HIF-1alpha-Mediated Telomerase Reverse Transcriptase Activation Inducing Autophagy Through Mammalian Target of Rapamycin Promotes Papillary Thyroid Carcinoma Progression During Hypoxia Stress. Thyroid (2021) 31:233–46. doi: 10.1089/thy.2020.0023 32772829

[42] Myllykangas S Buenrostro JD Natsoulis G Bell JM Ji HP. Efficient Targeted Resequencing of Human Germline and Cancer Genomes by Oligonucleotide-Selective Sequencing. Nat Biotechnol (2011) 29:1024–7. doi: 10.1038/nbt.1996 PMC4336783 22020387

[43] Alexander EK Kennedy GC Baloch ZW Cibas ES Chudova D Diggans J. Preoperative Diagnosis of Benign Thyroid Nodules With Indeterminate Cytology. N Engl J Med (2012) 367:705–15. doi: 10.1056/NEJMoa1203208 22731672

[44] Nikiforov YE Ohori NP Hodak SP Carty SE LeBeau SO Ferris RL. Impact of Mutational Testing on the Diagnosis and Management of Patients With Cytologically Indeterminate Thyroid Nodules: A Prospective Analysis of 1056 FNA Samples. J Clin Endocrinol Metab (2011) 96:3390–7. doi: 10.1210/jc.2011-1469 PMC3205883 21880806

[45] Pratilas CA Taylor BS Ye Q Viale A Sander C Solit DB. (V600E)BRAF Is Associated With Disabled Feedback Inhibition of RAF-MEK Signaling and Elevated Transcriptional Output of the Pathway. Proc Natl Acad Sci USA (2009) 106:4519–24. doi: 10.1073/pnas.0900780106 PMC2649208 19251651

[46] Kimura ET Nikiforova MN Zhu Z Knauf JA Nikiforov YE Fagin JA. High Prevalence of BRAF Mutations in Thyroid Cancer: Genetic Evidence for Constitutive Activation of the RET/PTC-RAS-BRAF Signaling Pathway in Papillary Thyroid Carcinoma. Cancer Res (2003) 63:1454–7. 12670889

[47] Suarez HG Du Villard JA Caillou B Schlumberger M Tubiana M Parmentier C. Detection of Activated Ras Oncogenes in Human Thyroid Carcinomas. Oncogene (1988) 2:403–6. 3283656

