# Supplementary material for: Mutation of Hashimoto’s Thyroiditis and Papillary Thyroid Carcinoma Related Genes and the Screening of Candidate Genes
Source: Front Oncol. 2021 Dec 21;11:813802. doi: 10.3389/fonc.2021.813802 (PMC8724914; doi:10.3389/fonc.2021.813802)
Supplement: Supplementary file 1 [file Table_1.docx]

**SUPPLEMENTARY TABLE S1** Sample numbering and grouping of chips

| ID | Group |
| --- | --- |
| GSM724489 | hashimoto thyroiditis |
| GSM724490 | hashimoto thyroiditis |
| GSM724491 | hashimoto thyroiditis |
| GSM724492 | hashimoto thyroiditis |
| GSM724493 | hashimoto thyroiditis |
| GSM724494 | hashimoto thyroiditis |
| GSM724512 | papillary thyroid carcinoma |
| GSM724513 | papillary thyroid carcinoma |
| GSM724514 | papillary thyroid carcinoma |
| GSM724515 | papillary thyroid carcinoma |
| GSM724516 | papillary thyroid carcinoma |
| GSM724517 | papillary thyroid carcinoma |
| GSM724518 | papillary thyroid carcinoma |
| GSM724519 | papillary thyroid carcinoma |
| GSM724520 | papillary thyroid carcinoma |
