# Supplementary figures and images for: Mutation of Hashimoto’s Thyroiditis and Papillary Thyroid Carcinoma Related Genes and the Screening of Candidate Genes
Source: Front Oncol. 2021 Dec 21;11:813802. doi: 10.3389/fonc.2021.813802 (PMC8724914; doi:10.3389/fonc.2021.813802)

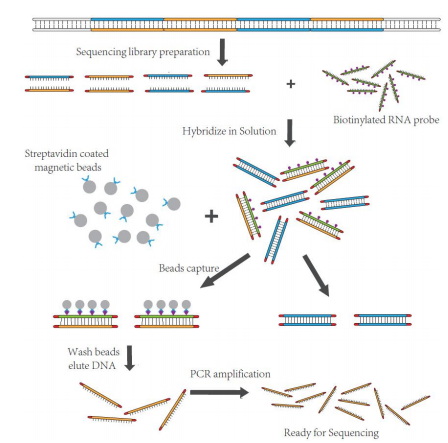

Supplement: Supplementary file 2 [file Image_1.tif]

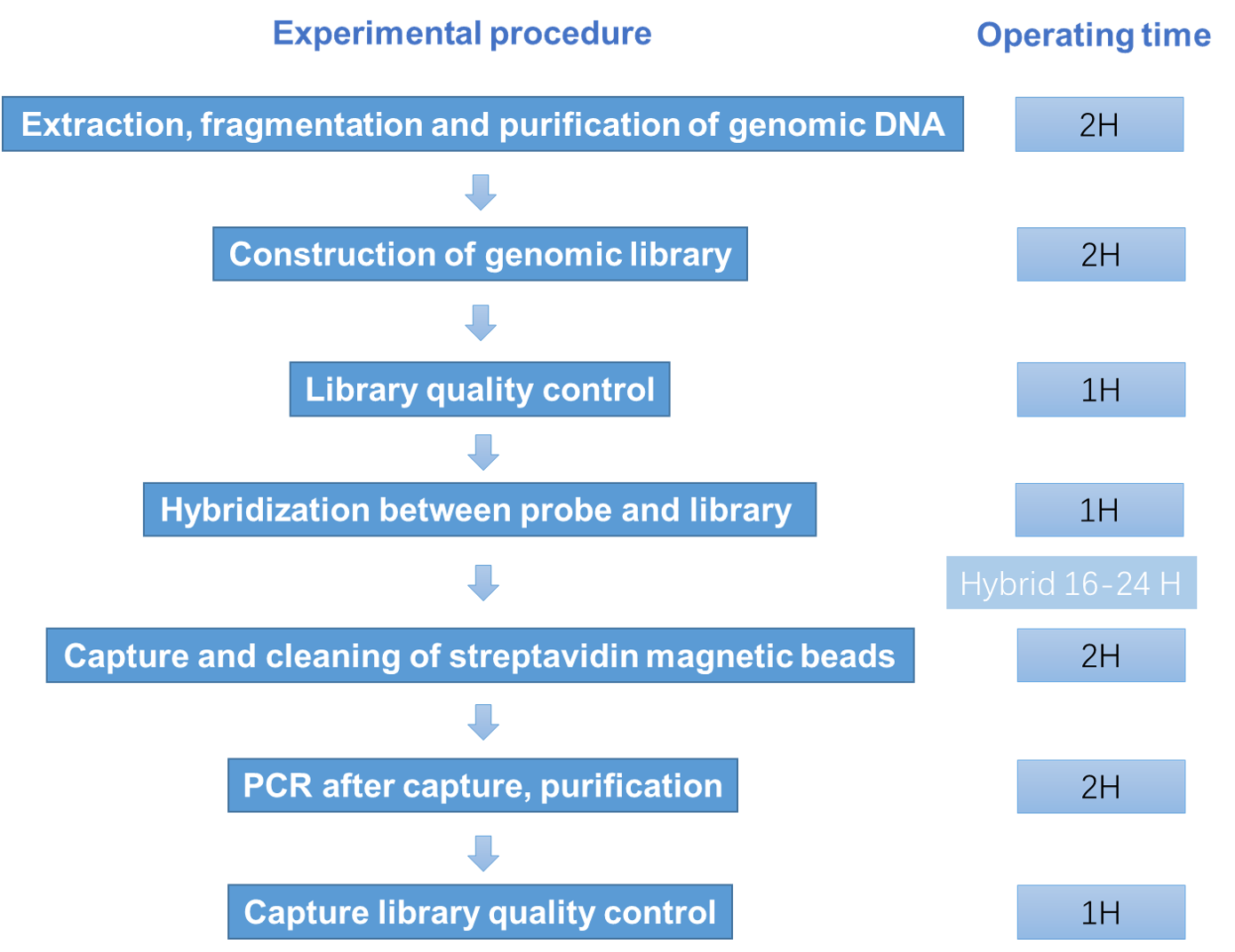

Supplement: Supplementary file 3 [file Image_2.tif]

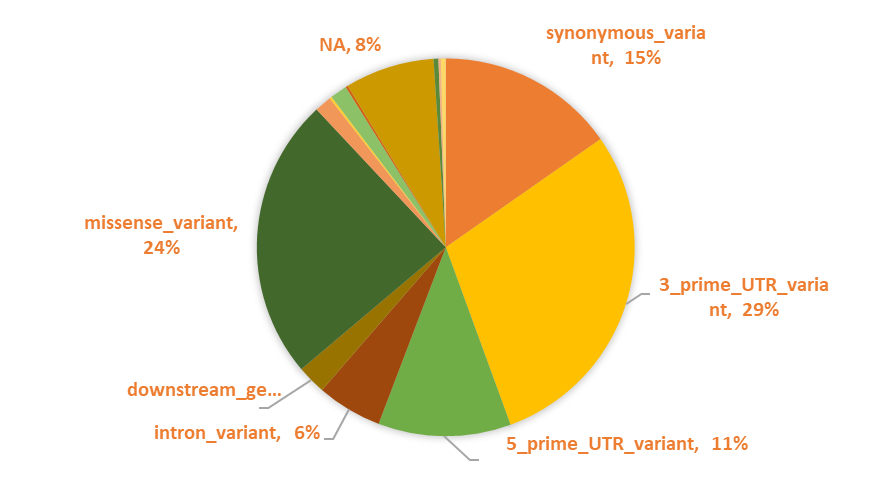

Supplement: Supplementary file 4 [file Image_3.tif]

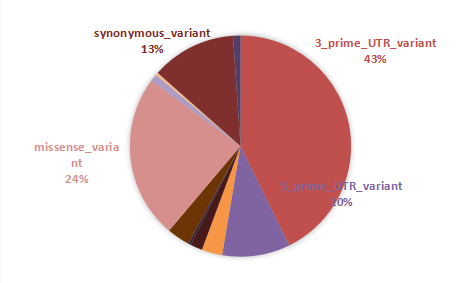

Supplement: Supplementary file 5 [file Image_4.tif]

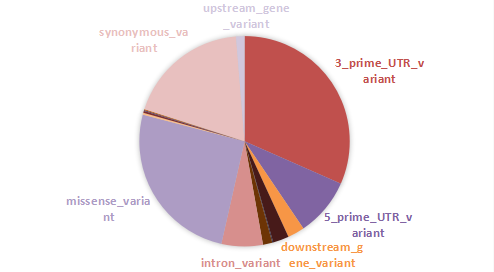

Supplement: Supplementary file 6 [file Image_5.tif]

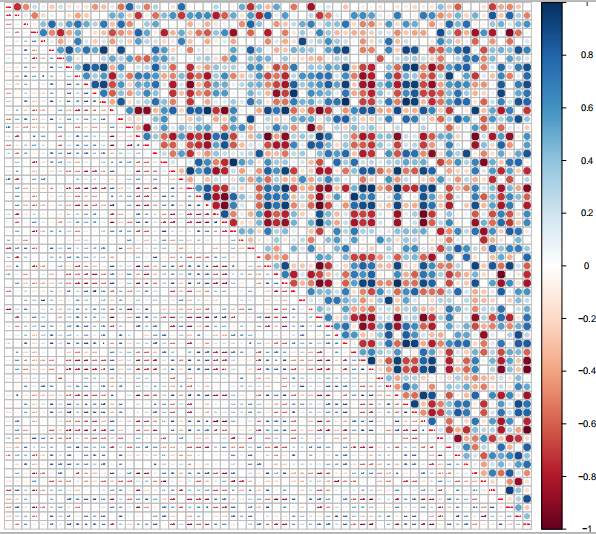

Supplement: Supplementary file 7 [file Image_6.tif]

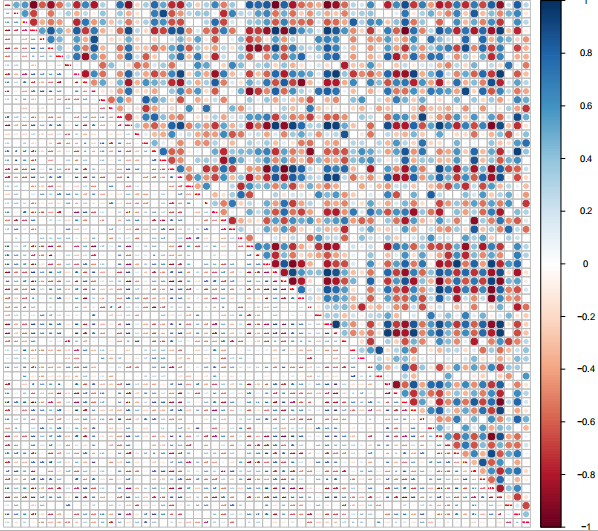

Supplement: Supplementary file 8 [file Image_7.tif]
